# Exploring the influence of rock bridge angle on the rock secant modulus

**DOI:** 10.1371/journal.pone.0307709

**Published:** 2024-09-25

**Authors:** Junxia Zhou, Shiyu Meng, Gaojian Hu, Lanchang Zha

**Affiliations:** 1 Ordos Institute of Liaoning Technical University, Ordos, China; 2 School of Civil Engineering, Liaoning Technical University, Fuxin, Liaoning Province, China; 3 School of Civil Engineering, Shaoxing University, Shaoxing, Zhejiang Province, China; University of Science and Technology Beijing, CHINA

## Abstract

Rock can undergo shape deformation and damage due to the influence of joint fissures, and the range of damage caused by joints at different rock bridge angles varies. To study the influence of rock bridge angle on the size effect of rock secant modulus, this paper adopts the principle of regression analysis and combines numerical simulation to carry out relevant research. The research results indicate that: (1) As the rock bridge angle increases, the secant modulus gradually decreases, following a power function relationship. (2) As the rock size increases, the secant modulus shows a trend of first decreasing and then stabilizing, following a power function relationship. (3) As the rock bridge angle increases, the characteristic tangent modulus and characteristic size gradually decrease, following a power function relationship. On this basis, we obtained their relationship formulas separately.

## 1.Introduction

Secant modulus (E_50_) refers to the ratio of stress to corresponding longitudinal strain in rock specimens under longitudinal stress. It is an indispensable parameter in rock mechanics research and plays an important role in rock mechanics theory. It is widely used in engineering design and rock stability analysis. The size of rocks and the geometric shape of internal cracks have a significant impact on their mechanical properties, which is reflected in the variation of E_50_. Secondly, the rock bridge angle is also one of the key indicators for evaluating rock stability, which also affects the E_50_. Therefore, in-depth research on the relationship between E_50_ and rock bridge angle is of great significance for accurately evaluating rock stability.

The E_50_, as an important parameter reflecting the deformation characteristics of rocks, has always been a hot topic in the field of rock mechanics’ research. Scholars have conducted in-depth research on the mechanical properties and deformation behavior of rocks within the nonlinear strain range through the study of E_50_. For example, Davarpanah et al. [1] explored the correlation between the critical mechanical properties of rocks through laboratory testing and statistical analysis and found a high correlation between the tangent value of E_50_ and Poisson’s ratio. Kang et al. [2] found that the decay rate of E_50_ decreases with increasing confining pressure. Jiang et al. [3] found that the E_50_ decreases with the axial stress based on the theory of continuous damage mechanics. Peng et al. [4] conducted a series of experiments on granite with different burial depths and found that the E_50_ and burial depth basically follows a quadratic function relationship. Peng et al. [5] found that as the strain rate increased, the E_50_ showed an increasing trend. Zong et al. [6] studied the effect of confining pressure and found that the E_50_ increases linearly with the confining pressure. Zhao [7] investigated the influence of prefabricated cracks on E_50_. Although these studies have explored the effects of confining pressure, burial depth, strain rate, and cracks on E_50_, they have rarely considered the influence of rock bridge angle and the impact of rock size changes. Therefore, to further understand the deformation characteristics of rocks, it is necessary to conduct in-depth research on the quantitative relationship between E_50_ and rock bridge angle.

Studying the relationship between E_50_ and size is an important topic in the field of rock mechanics. As research deepens, scholars have discovered some phenomena and patterns. For example, Liu et al. [8] studied the influence of joint length on rock mechanical properties using 3D printing technology and found that the E_50_ continued to decrease with the joint length. Zhu [9] explored the relationship between the E_50_ and size ratio of the sample based on the hardened soil model. Zhu et al. [10] investigated the effect of interface energy on E_50_ and found that the E_50_ is dependent on particle size. Koutou A et al. [11] found that the E_50_ is influenced by the material shape. Sun et al. [12] explored the influence of rock size on E_50_ using regression analysis. The above research mainly explores the effects of joint length, particle size, and particle shape on the E_50_, but there is little research on the influence of rock size on the E_50_, and the effect of rock bridge angle is also rarely considered.

Accurately determining the characteristic size of rocks is crucial in rock mechanics’ research. Representative Elemental Volume (REV) refers to a representative rock sample size that can provide accurate and statistically significant measurements of mechanical parameters at the minimum scale of macroscopic mechanical behavior. Previous studies have extensively explored the concept and applications of REV. For example, Huang et al. [13] validated and obtained an indicator for estimating rock mass REV based on GSI. Liu et al. [14] developed a method for estimating the geometric feature size REV of rocks based on rock anisotropy. Niazmandi et al. [15] studied the effect of constraints on the size of REV using the DFN to synthesize fractured rock masses. Zhou et al. [16] used three-dimensional laser scanning technology to study the influencing factors of REV. Ma et al. [17] studied the influence of crack size on rock masses REV. Wang et al. [18] proposed a REV estimation method based on permeability. These studies are mainly based on different indicator factors, such as GSI, anisotropy, permeability, etc., to estimate the REV value, with a focus on the definition and calculation methods of REV. However, little consideration is given to the properties of rock itself, such as rock size and angle.

Therefore, this article studies the influence of rock bridge angle on the E_50_, acquires their relationship, and obtains their influence on the characteristic size.

## 2. Numerical simulation scheme and boundary conditions

The numerical simulation scheme is carried out from the following two aspects. (1) Explore the relationship between the rock bridge angle and E_50_, and set the rock bridge angle to 15°, 30°, 45°, 60°, and 75°, including schemes 1–7. (2) Explore the relationship between rock size and E_50_ and set rock sizes as 100 mm to 400 mm, including schemes 8 to 12. The plans are shown in Table 1.

**Table 1:** Simulation schemes.

Simulation program	Size/mm	Scheme 8	Scheme 9	Scheme 10	Scheme 11	Scheme 12
		15°	30°	45°	60°	75°
Scheme 1	100	100×15°	100×30°	100×45°	100×60°	100×75°
Scheme 2	150	150×15°	150×30°	150×45°	150×60°	150×75°
Scheme 3	200	200×15°	200×30°	200×45°	200×60°	200×75°
Scheme 4	250	250×15°	250×30°	250×45°	250×60°	250×75°
Scheme 5	300	300×15°	300×30°	300×45°	300×60°	300×75°
Scheme 6	350	350×15°	350×30°	350×45°	350×60°	350×75°
Scheme 7	400	400×15°	400×30°	400×45°	400×60°	400×75°

In the simulation, the parameters of rock mechanics were obtained through geological surveys of rocks in the Haoba Mine in Shaoxing City. The parameters are shown in Table 2.

**Table 2:** Mechanical parameters.

Material	UCS/MPa	Elastic modulus/MPa	Poisson ratio	Internal friction/°
Rock	101.34	4874	0.25	48.32
Joint	0.01	0.01	0.25	40

**Fig 1:**
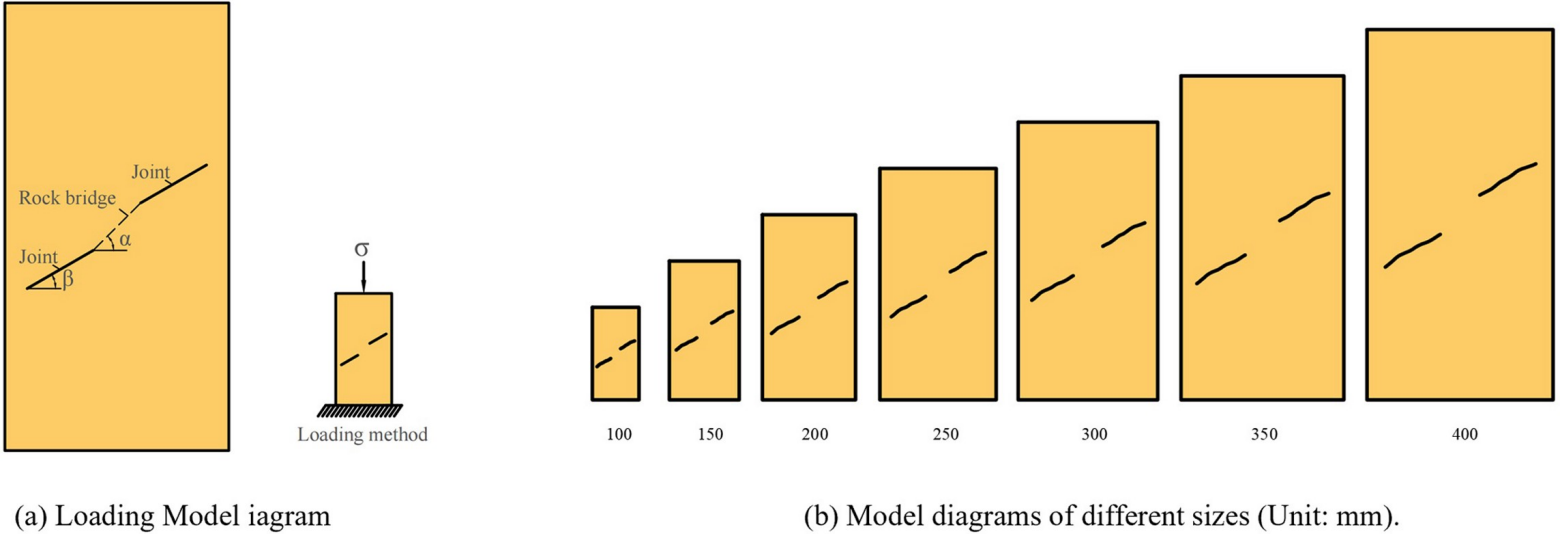
Calculation model diagram.

**Fig 2:**
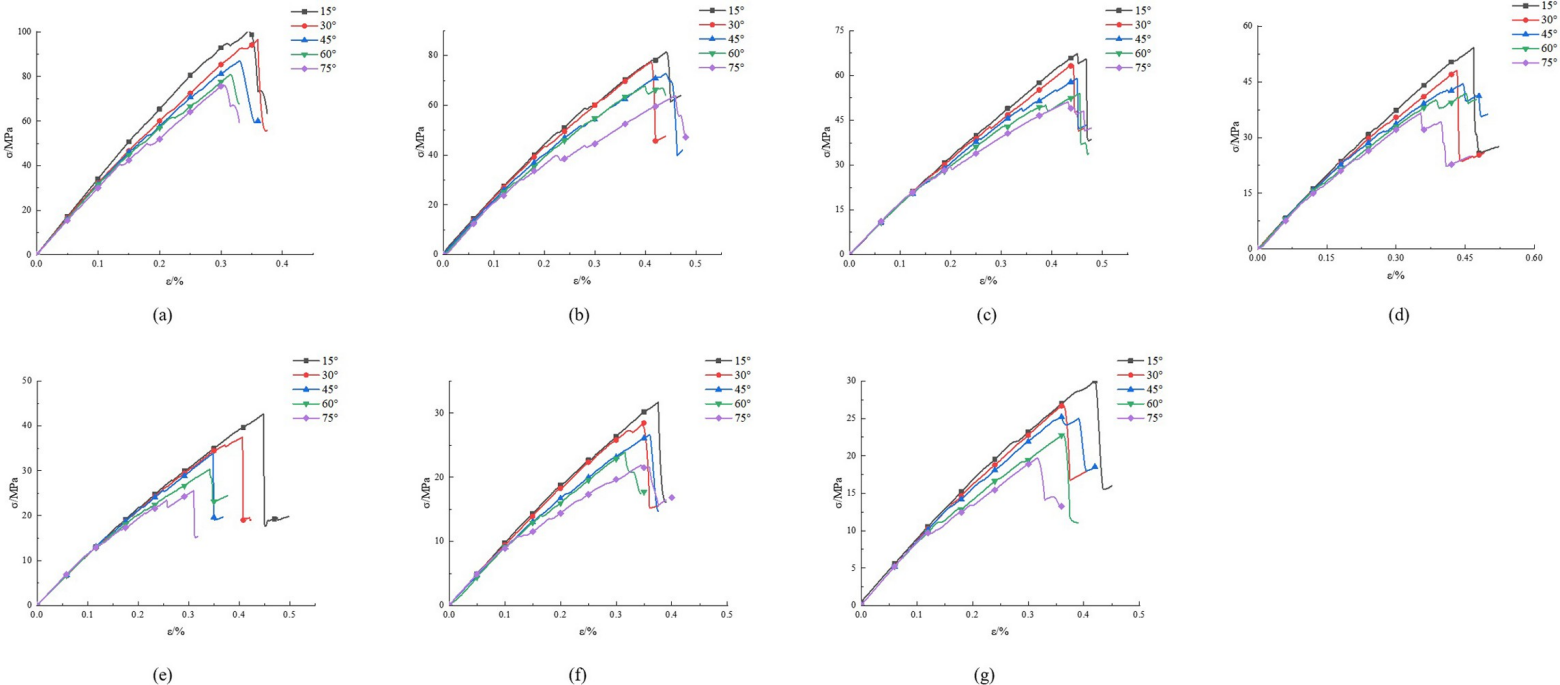
Stress-strain curves under different rock bridge angles. (a) 100 mm, (b) 150 mm, (c) 200 mm, (d) 250 mm, (e) 300 mm, (f) 350 mm, (g) 400 mm.

The loading model of this article is shown in Fig 1, where α is the rock bridge angle. The model adopts displacement loading, with an initial loading amount of 0 and a displacement amount of 0.01mm. The calculation models of different sizes are shown in Fig 2.

## 3. Result analysis

### 3.1 Analysis of stress-strain laws

In rock mechanics, the stress-strain curve of rocks provides the relationship between stress and strain, which can reveal the variation of the E_50_ of rocks. We conducted simulation studies on schemes 1 to 7 and obtained their stress-strain curves, as shown in Fig 2. We conducted simulation studies on schemes 8 to 12 and obtained their stress-strain curves, as shown in Fig 3.

**Fig 3:**
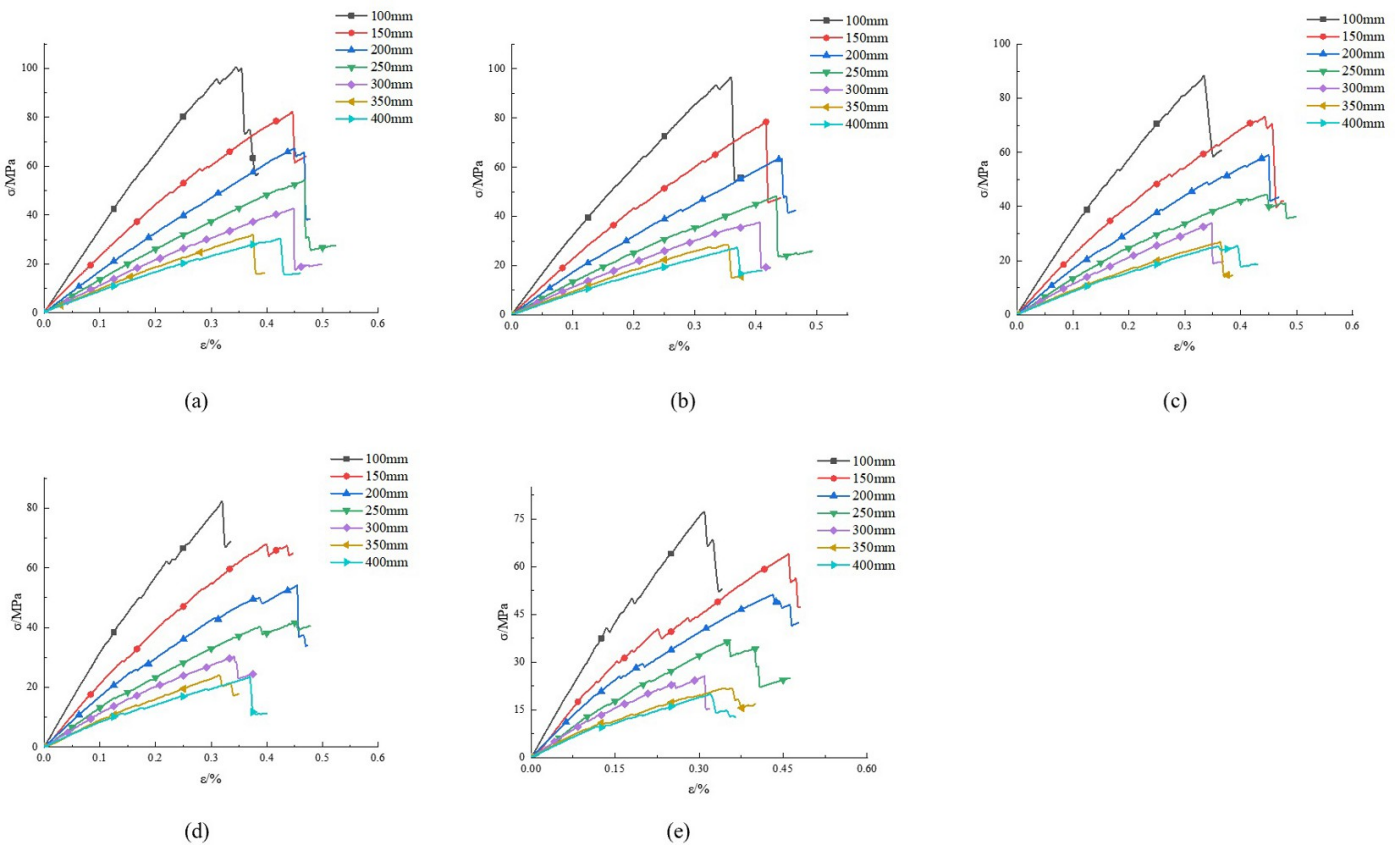
Stress-strain curves under different sizes. (a)15°, (b) 30°, (c) 45°, (d) 60°, (e) 75°.

Based on Figs 2 and 3, we select points corresponding to 50% compressive strength, and calculate the E_50_ value, as shown in Table 3.

**Table 3:** E_50_ values.

Simulation program	Size/mm	E_50_ /GPa				
		Scheme 8	Scheme 9	Scheme 10	Scheme 11	Scheme 12
		15°	30°	45°	60°	75°
Scheme 1	100	33.82	31.83	30.95	30.29	30.01
Scheme 2	150	24.68	22.63	21.72	21.22	20.91
Scheme 3	200	16.32	14.72	14.24	13.86	13.53
Scheme 4	250	12.95	11.84	11.23	10.93	10.69
Scheme 5	300	11.27	10.09	9.82	9.52	9.19
Scheme 6	350	9.75	8.41	7.81	7.58	7.26
Scheme 7	400	8.44	7.12	6.86	6.52	6.26

Analyzing Fig 2C, as the angle increases, the slope of the curve gradually decreases, and the calculated E_50_ also gradually decreases. When the angle varies between 15°, 30°, 45°, 60°, and 75°, the corresponding E_50_ values are 16.32 GPa, 14.72 GPa, 14.24 GPa, 13.86 GPa, and 13.53 GPa, respectively. Within the range of rock bridge angles from 15° to 75°, the E_50_ decreased by 17.09%. This phenomenon can be explained as the rock bridge angle causing a change in the stress distribution inside the rock. Normally, the E_50_ of rocks is controlled by the stress state inside the rock. When the rock bridge angle increases, the component of stress along the direction of the rock bridge decreases, while the normal component remains relatively stable. This will lead to a decrease in stress concentration, resulting in a decrease in the E_50_.

Analyzing Fig 3C, we found that the slope of the curve gradually decreases with increasing size. When the size increased from 100 mm to 300 mm, the E_50_ decreased from 30.95 GPa to 9.82 GPa, a decrease of 68.3%. After the size exceeds 300 mm, the E_50_ gradually stabilizes. This indicates that the E_50_ is significantly influenced by size. This is mainly due to the dominant characteristic factors of rock microstructure at smaller sample sizes. As the size increases, the dominant role of internal joint fractures in rocks strengthens, resulting in a gradual decrease in the E_50_. As the size increases, the proportion of internal joint cracks in the rock increases, and therefore the E_50_ gradually decreases.

In summary, the E_50_ decreases with the increase of rock bridge angle and rock size.

### 3.2 The effect of rock bridge angle on E_50_

**Fig 4:**
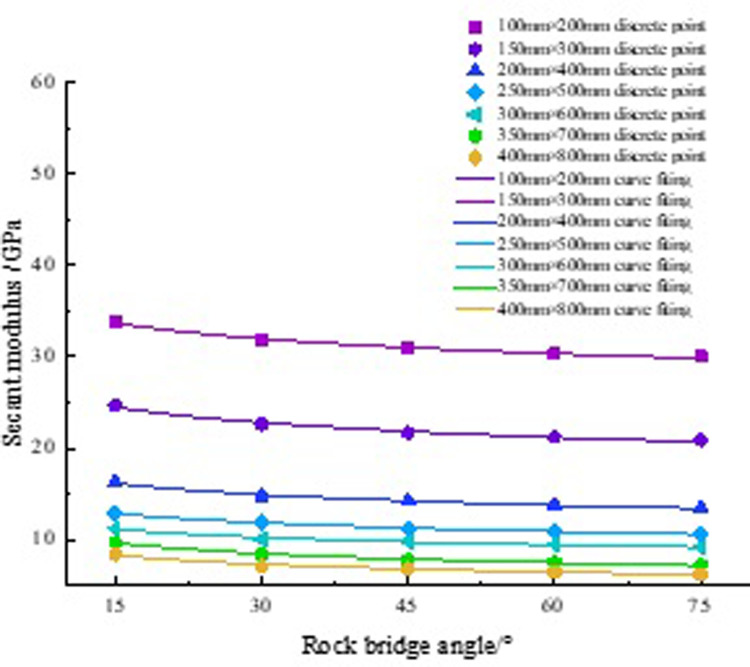
Fitting curve of E_50_.

The E_50_ decreases with the increase of the rock bridge angle, but their relationship still needs further exploration. Plot the curve of the change of E_50_ with the angle in Fig 4.

Fig 4 shows that each curve has two poles, namely the E_50_ at rock bridge angles of 15° and 75°. Analyzing the curve with a rock size of 350 mm, the E_50_ was the maximum value of the rock at a rock bridge angle of 15°, reaching 9.75 GPa. When the angle is 75°, its E_50_ is the minimum value, which is 7.26 GPa. During the process of increasing the angle from 15° to 75°, the E_50_ decreased by 25.54%. This indicates that as the angle increases, the E_50_ gradually decreases. Fit the relationship between them, as shown in Table 4.

**Table 4:** Fitting curve of rock E_50_.

Rock size/mm	Fitting formula	R^2^
100	$E_{50}\left(\alpha \right)=41.38\alpha^{-0.08}$	0.99
150	$E_{50}\left(\alpha \right)=32.61\alpha^{-0.1}$	0.98
200	$E_{50}\left(\alpha \right)=22.14\alpha^{-0.11}$	0.98
250	$E_{50}\left(\alpha \right)=17.91\alpha^{-0.12}$	0.99
300	$E_{50}\left(\alpha \right)=16.12\alpha^{-0.13}$	0.97
350	$E_{50}\left(\alpha \right)=15.41\alpha^{-0.18}$	0.99
400	$E_{50}\left(\alpha \right)=12.91\alpha^{-0.19}$	0.97

By analyzing the function types of these formulas, we found that they are all power functions. Based on this discovery, the following relationship between the rock bridge angle and E_50_ was proposed:

$$E_{50}\left(\alpha \right)=a\alpha^{b}$$ (1)

**Fig 5:**
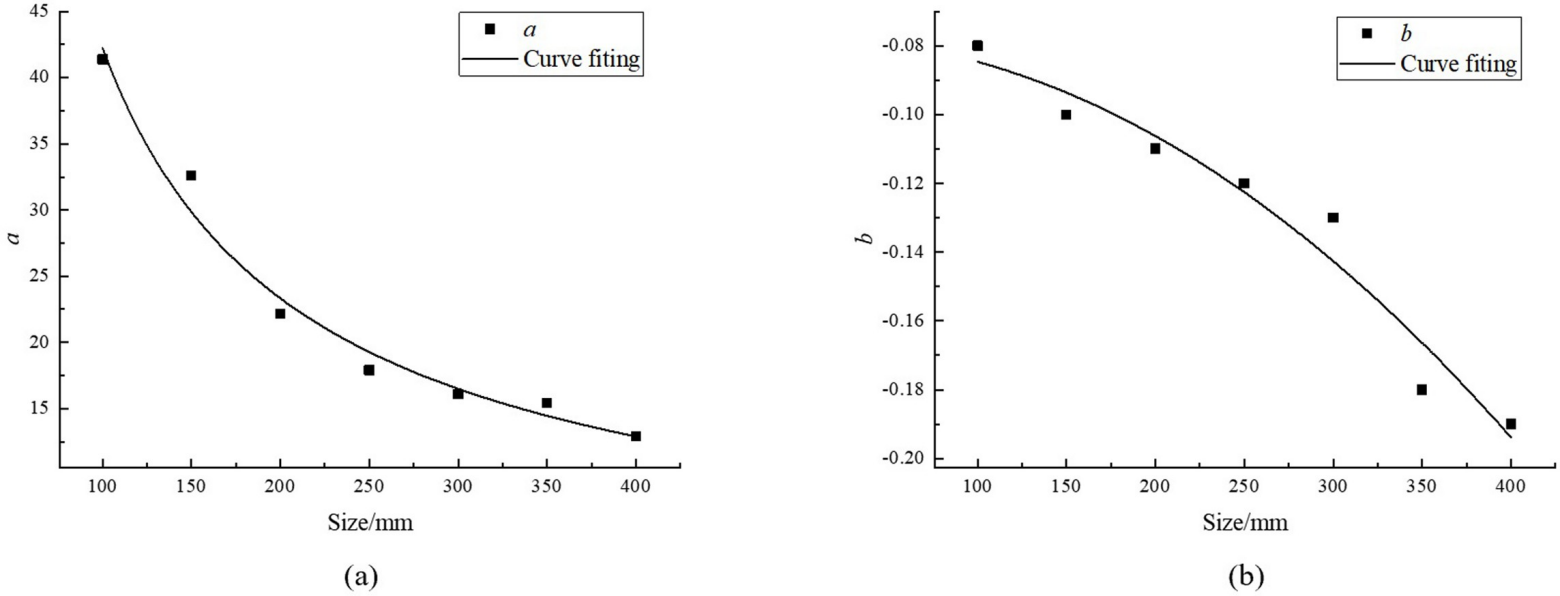
Fit curve graph. (a) a, (b) b.

In the formula, *E*_50_(*α*) is the E_50_, units: GPa; a, b are constant.

a and b are constants closely related to rock size. According to Table 4, the values of a and b were calculated, as shown in Table 5. Scatter plots of a and b with changes in rock size were plotted, and the curves were fitted in Fig 5.

**Table 5:** Constants a and b.

Size/mm Parameter	100	150	200	250	300	350	400
*a*	41.38	32.61	22.14	17.91	16.12	15.41	12.91
*b*	-0.08	-0.10	-0.11	-0.12	-0.13	-0.18	-0.19

Fig 5 shows that parameter a follows a power function relationship with rock size, and parameter b also follows a power function relationship with rock size, as follows:

$$a=2165.17l^{-0.86}$$ (2)

$$b=-0.08-6.34\times 10^{-7}\times l^{2.02}$$ (3)

From Eqs (1)-(3), we can obtain a special relationship for the E_50_ of rocks as follows:

$$E_{50}\left(\alpha \right)\text{=}2165.17l^{-0.86}\alpha^{-0.08-6.34\times 10^{-7}\times l^{2.02}}$$ (4)

Formula (4) is applicable for solving the E_50_ values of rocks of specific sizes. In this formula, the rock size is a known quantity. For a rock mass on site, when the rock bridge angle changes, we can calculate the corresponding E_50_ value.

### 3.3 The effect of rock size on E_50_

**Fig 6:**
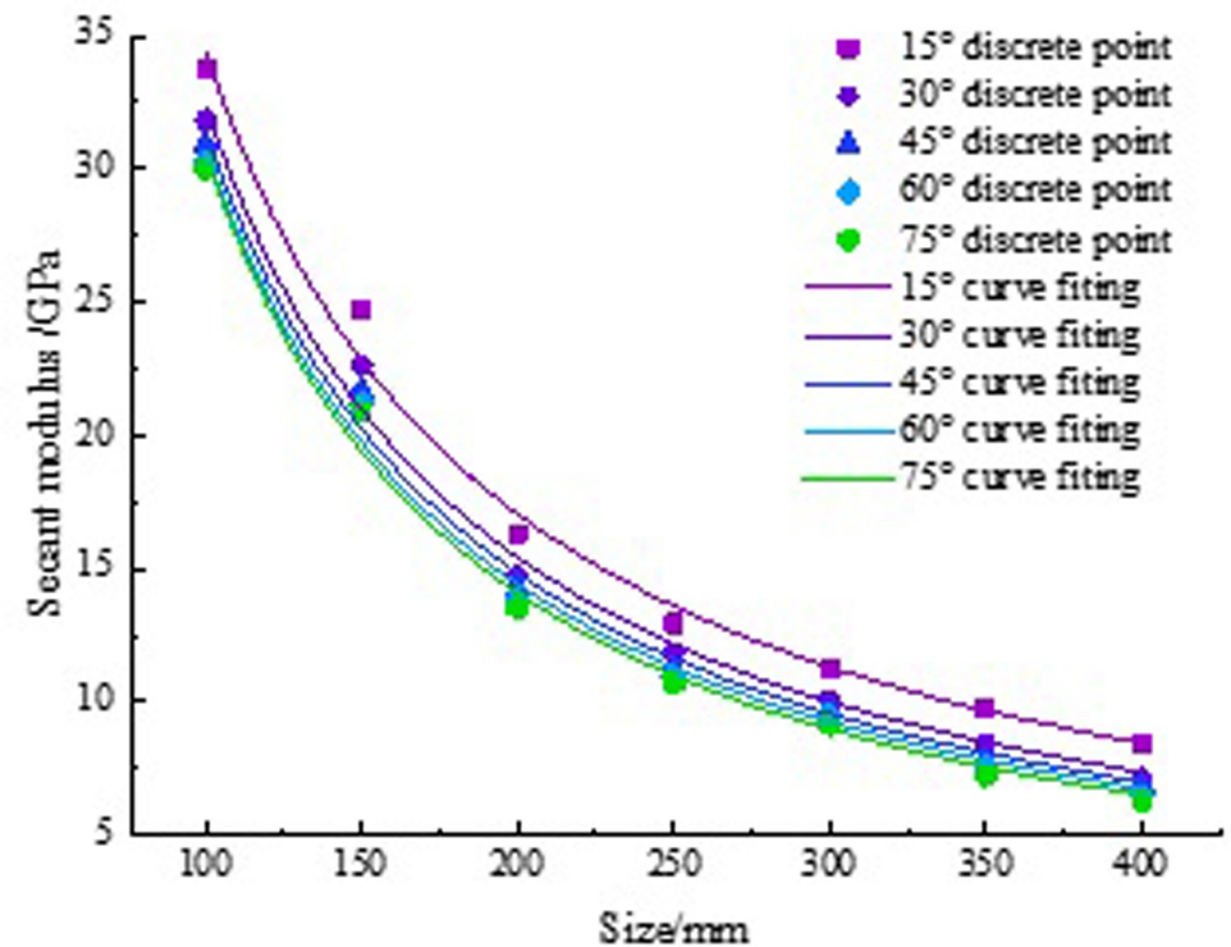
Fitting curves of E_50_.

Due to the size effect of rocks, changes in size will inevitably affect the E_50_. To obtain this effect, the relationship curves between E_50_ and rock size are plotted in Fig 6.

Analyzing Fig 6, as the size increases, the E_50_ decreases. When the rock size exceeds 350 mm, the E_50_ gradually tends to stabilize. This reflects the variation pattern of the pore structure inside the rock. At the beginning stage, as the rock size increases, the pore structure gradually increases. When the size reaches a certain threshold, the pore structure gradually tends to stabilize and no longer undergoes significant changes. Solve the fitting relationship for each curve in Table 6.

**Table 6:** Fitting relationship of E_50_.

Rock bridge angle/°	Fitting formula	Fitting coefficient (R^2^)
15	$E_{50}\left(l\right)=3638.61l^{-1.01}$	0.99
30	$E_{50}\left(l\right)=4425.49l^{-1.05}$	0.99
45	$E_{50}\left(l\right)=4616.88l^{-1.08}$	0.99
60	$E_{50}\left(l\right)=4925.74l^{-1.09}$	0.99
75	$E_{50}\left(l\right)=5154.94l^{-1.11}$	0.99

The formulas in Table 6 shows the relationships between E_50_ and rock size. Analyzing the function types of these formulas, we found that they all exhibit the characteristics of power functions. Based on this discovery, the following relationship was proposed:

$$E_{50}\left(l\right)=cl^{d}$$ (5)

**Fig 7:**
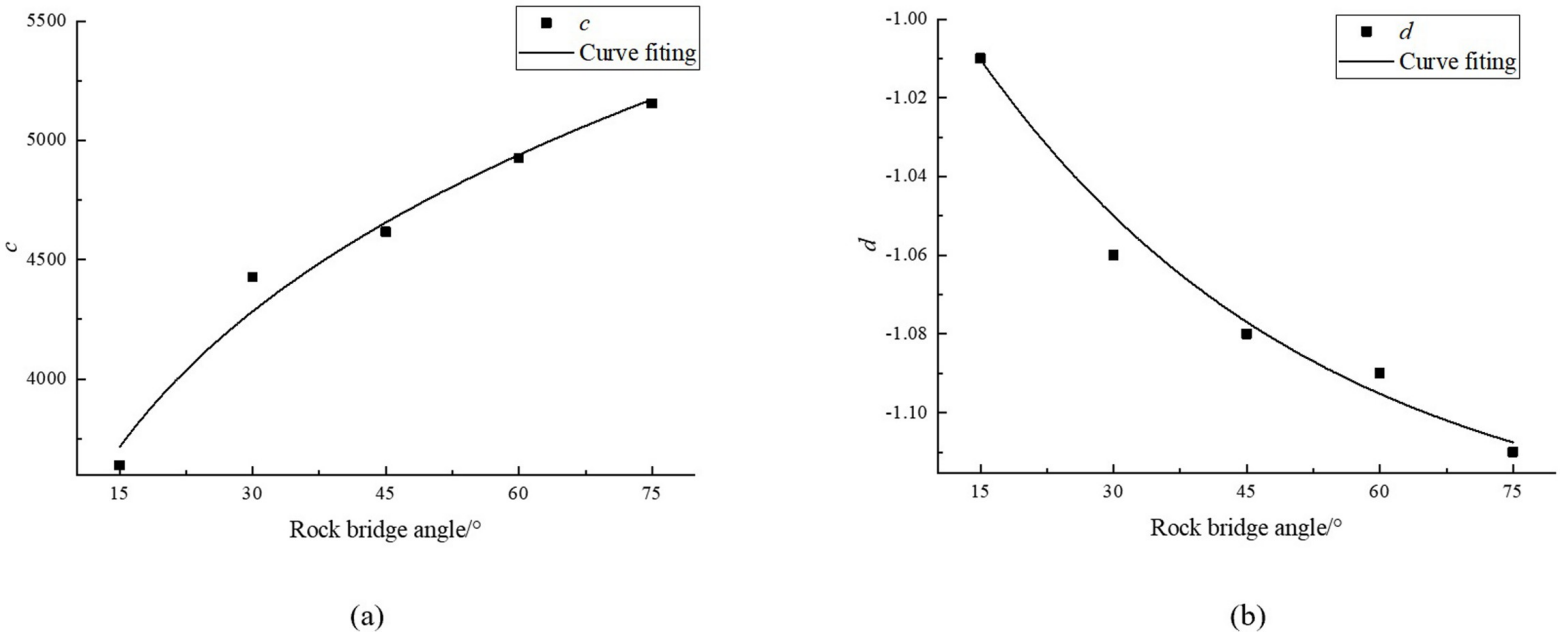
Fit curve graph. (a) c, (b) d.

In the formula, *E*_50_(*I*) is the E_50_, units: GPa.

c and d are constants closely related to the rock bridge angle. According to Table 6, the values of c and d under 5 rock bridge angles were calculated, as shown in Table 7. Scatter plots of c and d with the change of angle were plotted, and the curves were fitted in Fig 7.

**Table 7:** Parameters c and d.

Parameter	Rock bridge angle/°				
	15	30	45	60	75
*c*	3638.61	4425.49	4616.88	4925.74	5154.94
*d*	-1.01	-1.06	-1.08	-1.09	-1.11

Fig 7 shows that c and the angle follow a power function relationship, while d and the angle follow an exponential function relationship, as follows:

$$c=2128.51\alpha^{0.21}$$ (6)

$$d=0.18\times 0.97^{\alpha }-1.13$$ (7)

A special relationship for the E_50_ of rocks can be obtained from Eqs (5)-(7):

$$E_{50}\left(l\right)\text{=}2128.51\alpha^{0.21}l^{0.18\times 0.97^{\alpha }-1.13}$$ (8)

Formula (8) provides a method for solving the E_50_, in which the angle is a known quantity, and the rock size is a variable. For a rock, we can calculate the E_50_ value as it varies in size.

### 3.4 Fluctuation coefficient

Fluctuation coefficient reflecting the predictability of data and playing an important role in determining the engineering properties of rocks. Formula (9) provides the calculation formula for the fluctuation coefficient of the E_50_ [19]:

$$A_{l}=|\frac{E_{50l}-{\bar{E}}_{50l}}{{\bar{E}}_{50l}}|$$ (9)

Among them, A_*l*_ is the fluctuation coefficient; *E*_50*I*_ is the E_50_; ${\bar{E}}_{50l}$is the average value of the E_50_

**Fig 8:**
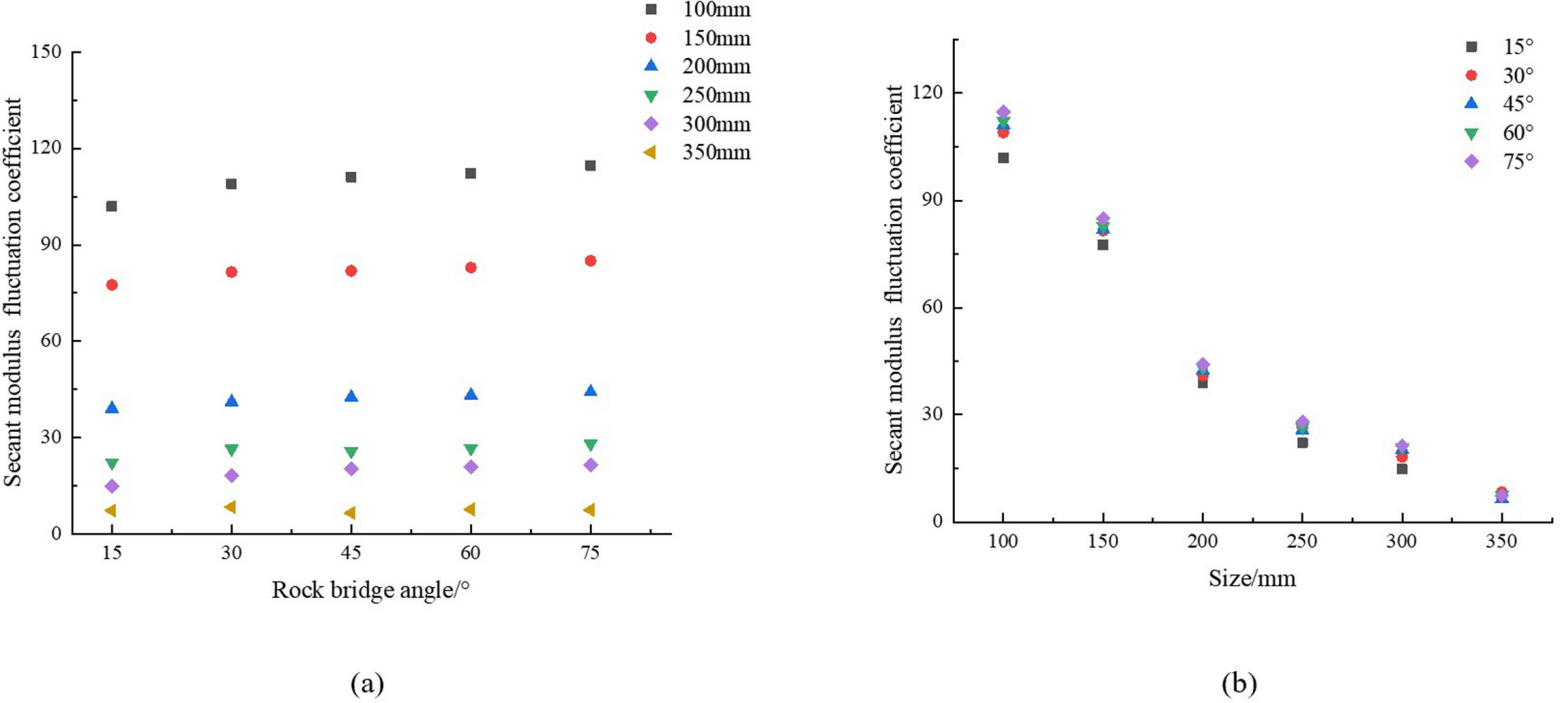
Variation law of E_50_ fluctuation coefficient (a) angle, (b) size.

We calculated the fluctuation coefficient of E_50_ according to formula (9), as shown in Table 8, and plotted the relationship curve in Fig 8.

**Table 8:** E_50_ fluctuation coefficient.

angle /° Size/mm	15	30	45	60	75
100	101.94	108.94	111.10	112.20	114.69
150	77.53	81.50	81.81	82.85	84.94
200	38.94	41.05	42.51	43.15	44.15
250	22.14	26.43	25.76	26.54	28.02
300	14.77	18.15	20.29	20.91	21.40
350	7.20	8.31	6.48	7.52	7.40

Analyzing Fig 8A, taking the rock size of 250 mm as an example, when the rock bridge angle increases from 15° to 75°, the fluctuation coefficient of E_50_ increases from 22.14 to 28.02, with an increase of up to 26.56%. This phenomenon indicates that as the angle increases, the fluctuation coefficient will increase.

Analyzing Fig 8B, as the rock size increases, the fluctuation coefficient of E_50_ decreases from 101.94 to 7.20, with a decrease of 92.94%. When the rock size reaches 350 mm, even if the rock bridge angle changes, the fluctuation coefficient is less than 10%, indicating that the E_50_ has stabilized.

### 3.5 Relationship between CSM and rock bridge angle

The characteristic size of rock secant modulus (CSSM) refers to the minimum size required for the determination of rock mechanics parameters, which has an impact on the study of rock mechanical properties. Reference [20] provides a calculation method for characteristic size.


$$\left|k|=|cdl^{d-1}\right|$$ (10)

|k|≤γ (11)

$$l\geq \sqrt[{d-1}]{\left|\frac{\gamma }{cd}\right|}$$ (12)

In the formula: γ is the absolute value of the slope. $\sqrt[{d-1}]{\left|\frac{\gamma }{cd}\right|}$can be considered as a formula for solving CSSM.

#### 3.5.1 CSSM and rock bridge angle

**Fig 9:**
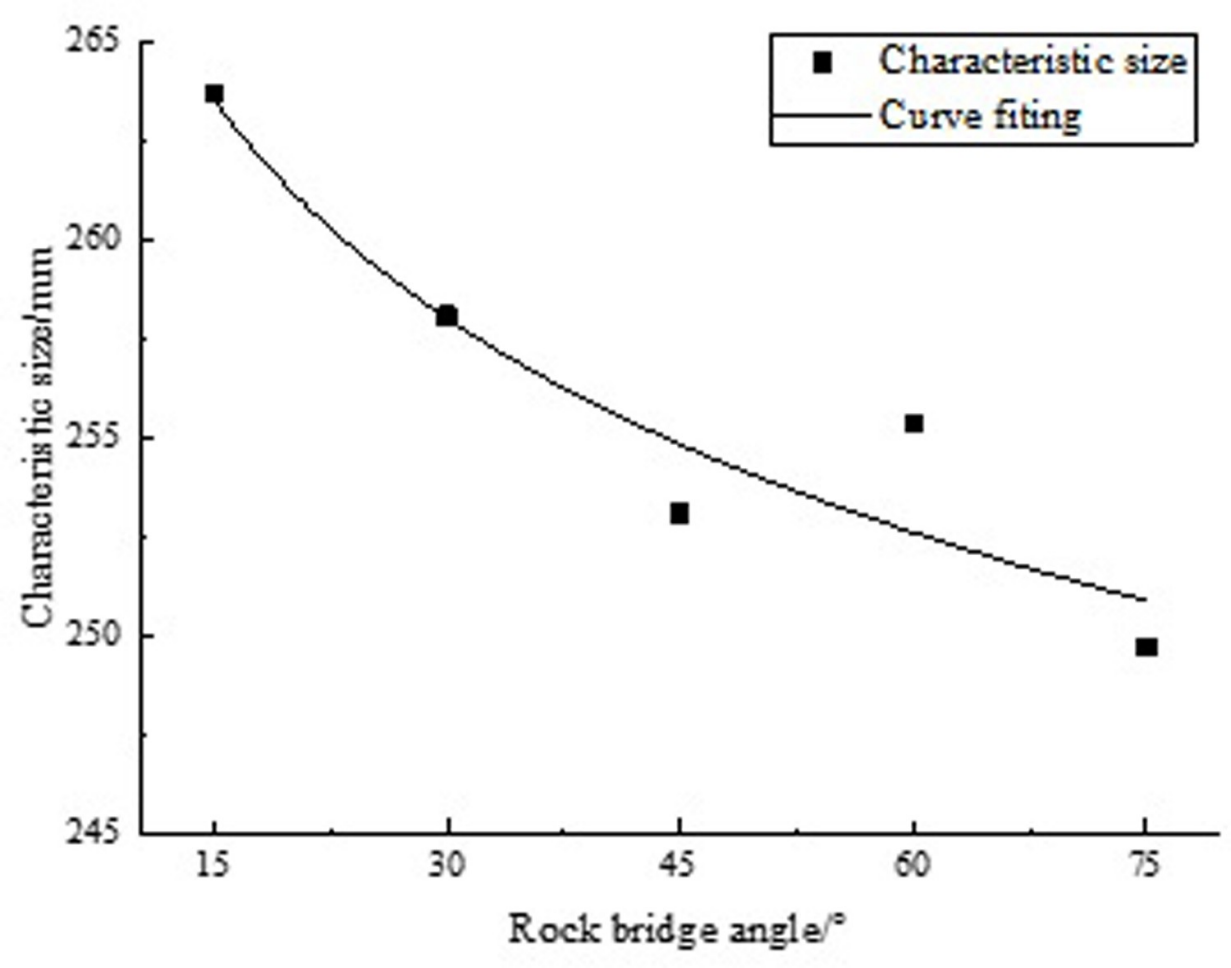
Fitting curve between CSSM and angle.

According to formula (12), we calculated the CSSM for different angles, as shown in Table 9. To clearly observe the trend and pattern of rock CSSM changes under different angles, their scatter plots and fitting curves were plotted in Fig 9.

**Table 9:** Characteristic size values.

angle/°	15	30	45	60	75
CSSM /mm	263.66	258.07	253.09	255.35	249.71

By analyzing Fig 9, the following conclusion can be drawn: as the angle increases, the CSSM shows a gradually decreasing trend. We conducted data fitting analysis on the collected data and found that they have a power function form, as follows:

$$D(\alpha )=286.21\alpha^{-0.03}$$ (13)

In the formula: *D*(*α*) is the CSSM, units: mm.

Formula (13) is used to calculate the CSSM, which is related to the rock bridge angle. But only applicable to rock bridges with two rough joints. The acquisition of this formula makes it convenient and fast to solve the CSSM on site.

#### 3.5.2 CSM and rock bridge angle

**Fig 10:**
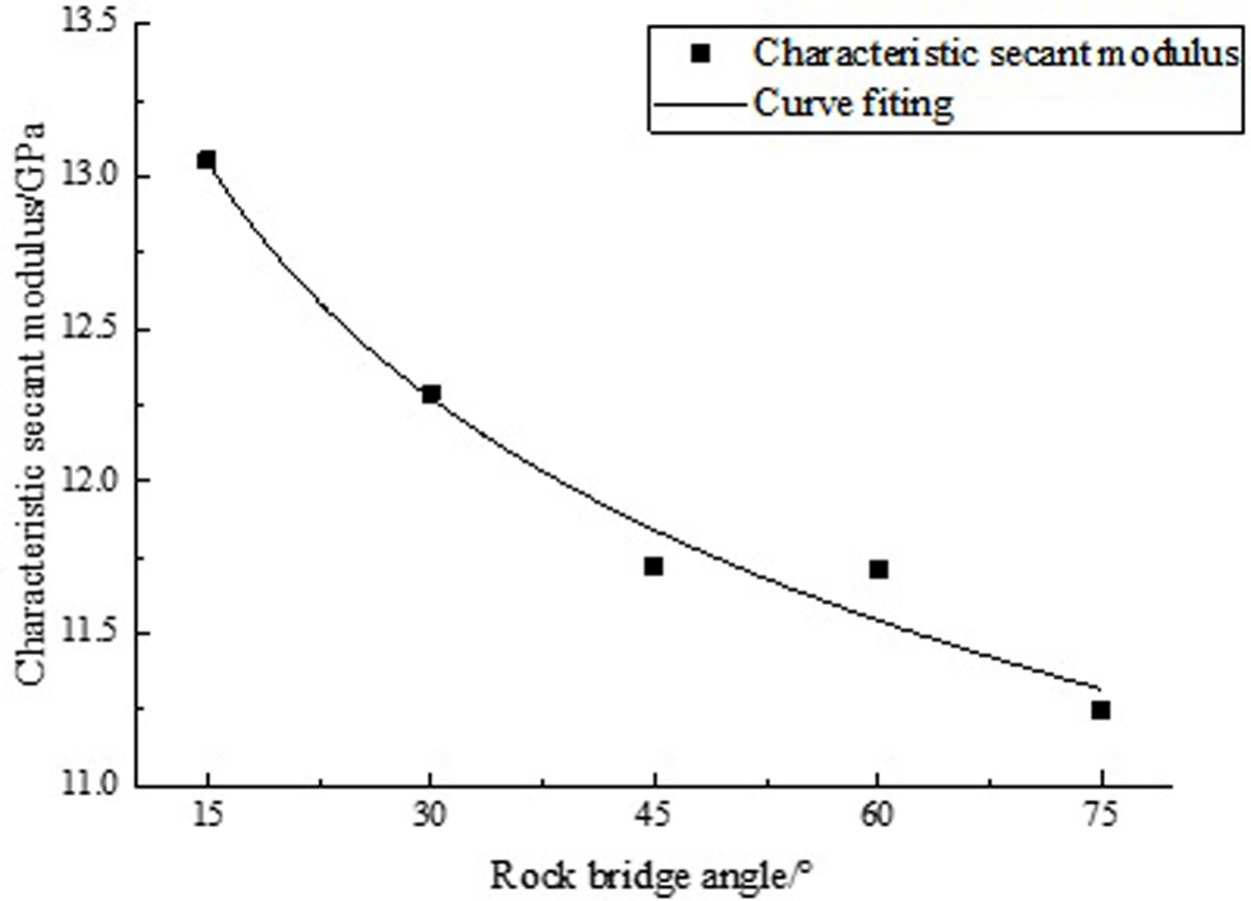
Fitting curve of CSM and rock bridge angle.

Based on the relevant data in Table 9, the characteristic secant modulus (CSM) values in Table 10 were obtained by formula (5), and the relationship was fitted in Fig 10.

**Table 10:** CSM.

angle/°	15	30	45	60	75
CSM /GPa	13.05	12.29	11.72	11.71	11.25

According to Fig 10, as the rock bridge angle increases, the CSM gradually decreases. This decreasing trend can be described by a power function, the regression formula is derived:

$$E_{w}(\alpha )=16.57\alpha^{-0.09}$$ (14)

In the formula, *E*_*w*_(*α*) is the CSM, units: GPa.

Formula (14) can be used to solve the CSM of rocks, which is related to the rock bridge angle. But it is only applicable for calculating rock bridges with two rough joints.

### 3.6 Validation analysis

**Fig 11:**
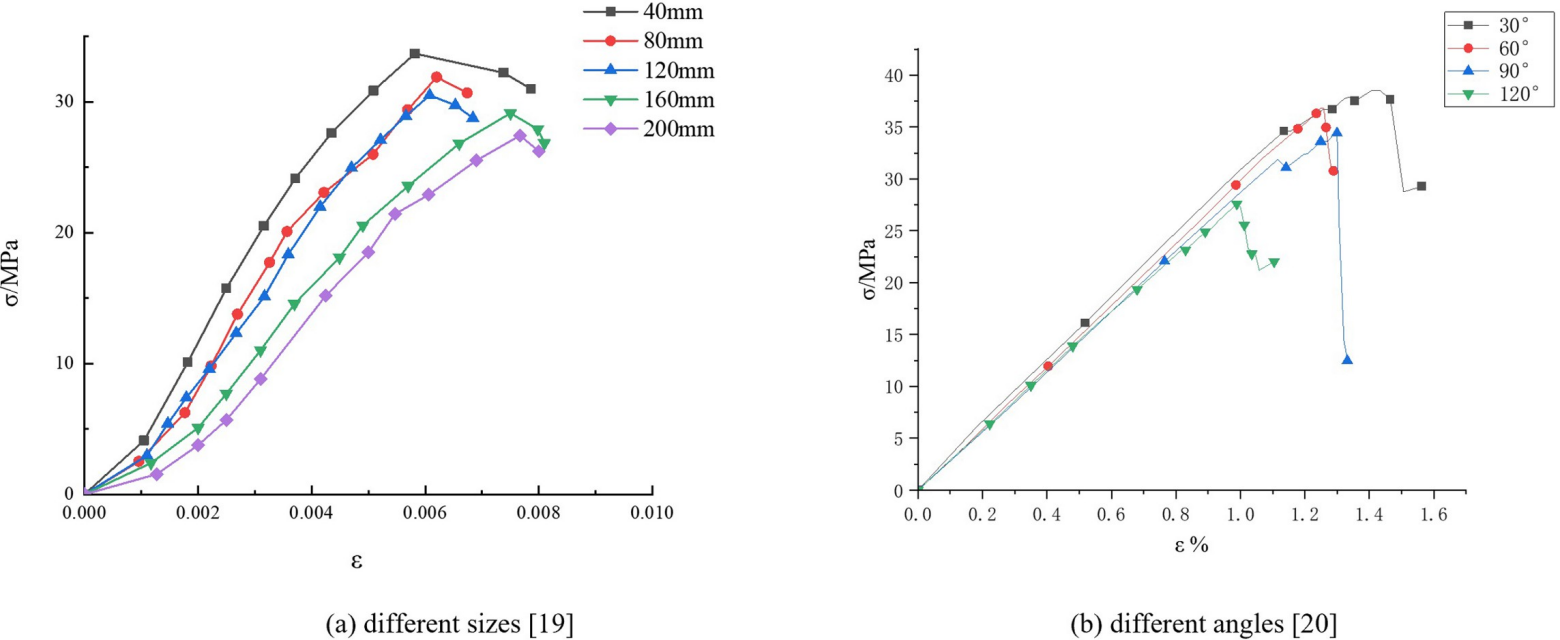
stress-strain curves.

To verify the accuracy of formulas (1) and (5), this section cites stress-strain curve data for different sizes and angles of references [19,21], as shown in Fig 11. Based on Fig 11, the E_50_ value is calculated, as shown in Tables 11 and 12.

**Table 11:** E_50_ values of size.

Size/mm	40	80	120	160	200
E_50_/GPa	6.01	5.32	4.77	3.83	3.43

**Table 12:** E_50_ values of angle.

angle/°	30	60	90	120
E_50_/GPa	31.04	30.18	29.45	28.14

**Fig 12:**
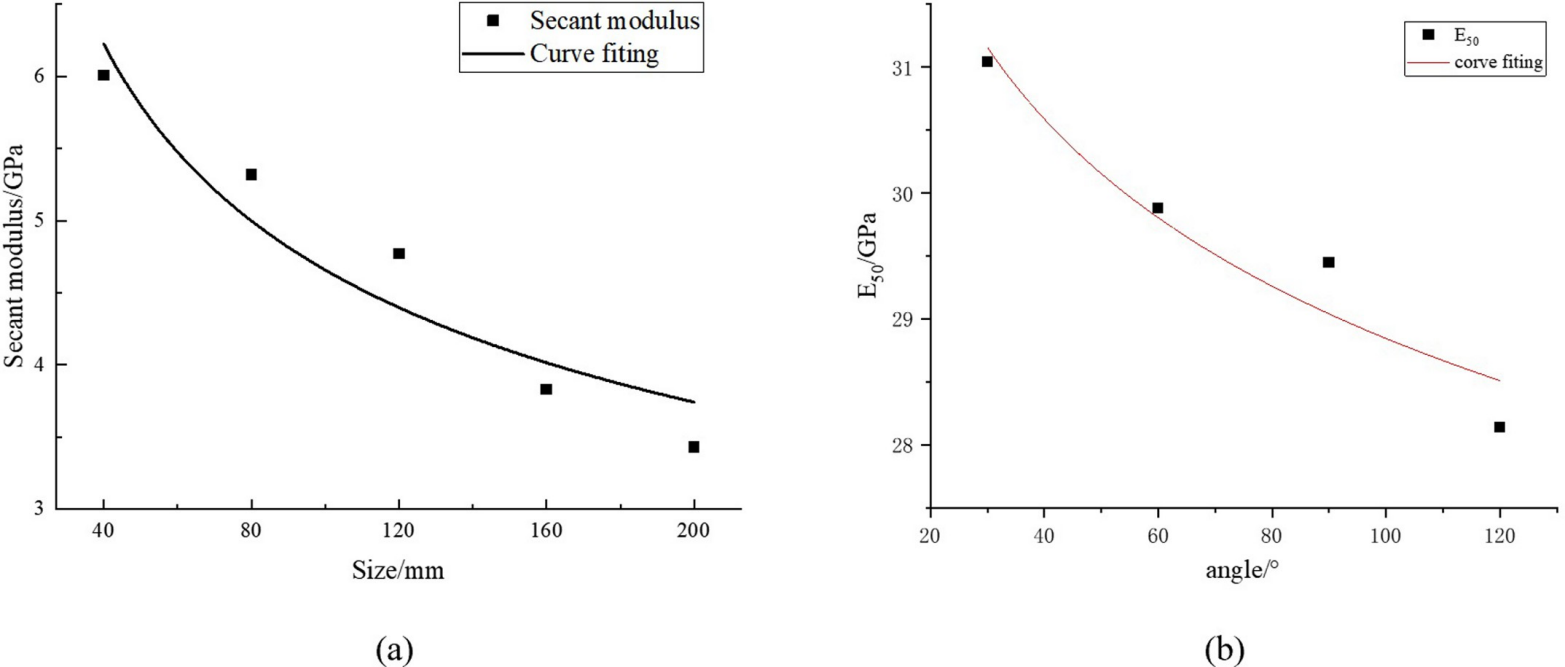
Curve fitting of E_50_.

The relationship curve between size and E_50_, as well as angle and E_50_, were fitted and plotted in Fig 12.

Through linear regression analysis of Fig 12, the relationship between size and E_50_, as well as angle and E_50_, were obtained as follows:

$$E_{50}\left(l\right)=19.97l^{-0.32}$$ (15)

$$E_{50}\left(\alpha \right)=38.73\alpha^{-0.064}$$ (16)

The function types of the formulas proposed in formula (15) and formula (5) are consistent, as well as formula (16) and formula (1), which proves the accuracy of formulas (5) and (1) and verifies the accuracy of the analysis method in this article.

## 4. Discussion

This article establishes three relationships:

1. The relationship between E_50_ and angle. scholars mostly explore the effects of confining pressure [6], burial depth [4], and strain rate [5] on the E_50_, but rarely discuss the relationship between angle and E_50_. Therefore, the relationship between angle and E_50_ obtained in this article provides important help for us to deeply understand the influence of rock bridge angle on E_50_.
2. The relationship between E_50_ and rock size. In existing studies, scholars have mostly studied the effects of joint length [8], particle size ratio [9], and particle shape [11] on the E_50_, with little consideration given to the influence of angle on size changes. The relationship obtained in this article provides us with assistance in gaining a deeper understanding of the size effect of E_50_.
3. The relationship between the CSM and the angle. This relationship is established based on obtaining the CSSM. In existing research, there has been relatively little research on the CSSM and CSM, and there has been little discussion on the relationship between CSM and angle. The relationship obtained in this article compensates for this deficiency.

## 5. Conclusion

The size effect exists in the E_50_ and the presence of rock bridge angle can also affect the results of size effect. This study investigated this issue through numerical simulation and drew the following conclusions:

(1) The relationship between the rock bridge angle and E_50_ is:

$$E_{50}\left(\alpha \right)=a\alpha^{b}$$

By solving the a and b, a specific formula was obtained as follows:

$$E_{50}\left(\alpha \right)\text{=}2165.17l^{-0.86}\alpha^{-0.08-6.34\times 10^{-7}\times l^{2.02}}$$

(2) The relationship between rock size and E_50_ is:

$$E_{50}\left(l\right){\text{=cl}}^{d}$$

The special relationship was obtained by solving the c and d as follows:

$$E_{50}\left(l\right)\text{=}2128.51\alpha^{0.21}l^{0.18\times 0.97^{\alpha }-1.13}$$

(3) The CSSM is related to the rock bridge angle, and the following specific forms are given based on the fitting:

$$D(\alpha )=286.21\alpha^{-0.03}$$

(4) The CSM is related to the rock bridge angle, and the following specific forms are given based on fitting:

$$E_{w}(\alpha )=16.57\alpha^{-0.09}$$
